# A comparison of Percutaneous femoral access in Endovascular Repair versus Open femoral access (PiERO): study protocol for a randomized controlled trial

**DOI:** 10.1186/s13063-015-0911-y

**Published:** 2015-09-14

**Authors:** Bastiaan P. Vierhout, Ben R. Saleem, Alewijn Ott, Jan Maarten van Dijl, Ties D. van Andringa de Kempenaer, Maurice E. N. Pierie, Jan T. Bottema, Clark J. Zeebregts

**Affiliations:** Department of Surgery, Wilhelmina Ziekenhuis Assen, Europaweg-Zuid 1, 9401 RK Assen, The Netherlands; Department of Surgery (Division of Vascular Surgery), University Medical Center Groningen, University of Groningen, Groningen, The Netherlands; Department of Microbiology, CERTE, Groningen, The Netherlands; Department of Medical Microbiology, University Medical Center Groningen, University of Groningen, Groningen, The Netherlands; Department of Surgery, Medical Center Leeuwarden, Leeuwarden, The Netherlands; Department of Vascular Surgery, Isala Hospital, Zwolle, The Netherlands; Department of Surgery, University Medical Center Groningen, University of Groningen, Groningen, The Netherlands

## Abstract

**Background:**

Access for endovascular repair of abdominal aortic aneurysms (EVAR) is obtained through surgical cutdown or percutaneously. The only devices suitable for percutaneous closure of the 20 French arteriotomies of the common femoral artery (CFA) are the Prostar™ and Proglide™ devices (Abbott Vascular). Positive effects of these devices seem to consist of a lower infection rate, and shorter operation time and hospital stay. This conclusion was published in previous reports comparing techniques in patients in two different groups (cohort or randomized). Access techniques were never compared in one and the same patient; this research simplifies comparison because patient characteristics will be similar in both groups.

**Methods/Design:**

Percutaneous access of the CFA is compared to surgical cutdown in a single patient; in EVAR surgery, access is necessary in both groins in each patient. Randomization is performed on the introduction site of the larger main device of the endoprosthesis. The contralateral device of the endoprosthesis is smaller. When we use this type of randomization, both groups will contain a similar number of main and contralateral devices. Preoperative nose cultures and perineal cultures are obtained, to compare colonization with postoperative wound cultures (in case of a surgical site infection). Furthermore, patient comfort will be considered, using VAS-scores (Visual analog scale). Punch biopsies of the groin will be harvested to retrospectively compare skin of patients who suffered a surgical site infection (SSI) to patients who did not have an SSI.

**Discussion:**

The PiERO trial is a multicenter randomized controlled clinical trial designed to show the consequences of using percutaneous access in EVAR surgery and focuses on the occurrence of surgical site infections.

**Trial registration:**

NTR4257 10 November 2013, NL44578.042.13.

## Background

Percutaneous endovascular abdominal aortic aneurysm repair (pEVAR) has been stated to be safe and feasible [1], even though several publications report a substantial number of complications [2–4]. With evolving experience, various risk factors have been identified, including obesity, groin scarring, common femoral artery (CFA) morphology and tortuous iliac arteries [1, 5].

Many current devices successfully close percutaneous punctures of the CFA up to 8 Fr.: such as the Angio-Seal™ (St. Jude Medical, Minnetonka, MN, USA), Mynx™ (AccessClosure, Mountain View, CA, USA), and the Starclose™ device (Abbott Vascular, Redwood City, CA, USA). The Prostar XL™ device (Abbott Vascular) has been used in femoral arteries, after sheaths of 18 to 24 Fr., with high rates of procedural success [1]. Wound infections appear to be absent [3, 6, 7] or few (0.2 %) [8]. In contrast, open femoral access has wound infection rates varying from 6.4 % [9] to 7.3 % [7]. Also, a longer surgery and hospital stay have been reported [10–12]. In percutaneous access, secondary interventions were more commonly seen, compared to surgical cutdown [10, 11]. Still, in suitable patients, this seems to be the only device capable of closing a 20 Fr. percutaneous arterial access site.

Although research has been performed, comparing both techniques, a comparison of both techniques in one patient has never been performed prior to now. Risk factors (obesity, CFA morphology and tortuosity of the iliac arteries) will be equally divided in this study design. Therefore, postoperative complications will mainly depend on the technique used and on the diameter of the device implanted. Also, each patient will be able to compare the result of both techniques by subjective pain sensation or discomfort.

## Methods/Design

### Study design

In the current study, percutaneous femoral access is compared to open femoral access. Bilateral femoral access is required in each patient treated with a bifurcated EVAR. Therefore, each patient can serve as his/her own control. Randomization is performed for the caliber of the endovascular device (main device versus contralateral extension) that is introduced within the aorta.

The PiERO trial is designed as a randomized, controlled, nonblinded, multicenter superiority trial with two parallel groups and a primary endpoint of wound infection during the 30 days after surgery. Simple randomization will be performed concerning the diameter of the device component introduced in the artery, with a 1:1 allocation.

### Sample size calculation

The assumption has been made that a pEVAR is less likely to cause a surgical site infection (SSI) than an EVAR with surgical cutdown to the femoral artery. We estimated the number of patients needed in a superiority trial with an effect size of 90 % and a margin of 10 (alpha 5 %, power 80 %). Assuming an SSI incidence of 6.8 % (Table 1) in an open surgical femoral cutdown and of 0.1 % in pEVAR, where *p*1 = 0.068 and *p*2 = 0.001 and using the equation *n* = 8 × (*p*1(1 − *p*1) + *p*2(1 − *p*2))/(*p*1 − *p*2)^2^ [13], 120 patients are required to reach sufficient evidence and avoid a type 2 error (including a fall-out percentage of 5 %).

**Table 1:** Wound complication rates mentioned in various studies

Studies	Surgical cutdown	Percutaneous access	Number of patients	*P* value	Research type
Johanning (2001) [8]	nm	0.2	489	-	retrospective
Nehler (2001) [3]	nm	0	189	-	systematic review
Eisenack (2009) [6]	nm	0	500	-	prospective
Zhong (2011) [7]	7.3	0	236	<0.01	retrospective
RIVM (2014)	6.4	nm	329	-	retrospective
Total:	6.8	0.1	1743		

*nm* not mentioned

### Setting

The trial is a multicenter trial, including one academic hospital and four general hospitals in the northern part of the Netherlands. In each center, all vascular surgeons perform surgeries for this study (Table 2). Participating hospitals are outlined on www.pierotrial.nl. In participating centers, each surgeon or interventionalist performing the procedure must have had the experience of 20 Prostar™procedures or 10 Proglide™ procedures prior to including patients in the PiERO trial, to prevent a performance bias. The Proglide™ procedure is a procedure easier to master. A minimum experience of 20 surgical cut downs to the common femoral artery (CFA) is also required. All punctures of the CFA should be performed with ultrasound guidance.

**Table 2:** Participating centers, local judging committees and number of surgeons

Participating center	Local medical ethical committee	Number of surgeons
Isala Hospital	METC Isala	four vascular surgeons
	Dr. van Deenweg 1 to 11	
	Zwolle	
Medical Centre of Leeuwarden	MCL Academie	three vascular surgeons
	Borniastraat 34	
	Commissie onderzoeksverklaring	
	Leeuwarden	
Scheper Hospital Emmen	METc Scheper Hospital	two vascular surgeons
	Boermarkeweg 60	
	Emmen	
University Medical Center of Groningen	METc UMCG	five vascular surgeons
Wilhelmina Hospital Assen	Board of Governance	two vascular surgeons
	Europaweg-Zuid 1	
	Assen	

### Primary endpoints

The primary endpoints include the number of SSI’s in the groin within 30 days postoperatively and at final evaluation 1 year after the operation. Wounds will be evaluated by means of the Southampton Wound Assessment score [14]. This score lowers the variability in wound assessment by various researchers. When an SSI is diagnosed, both clinically and through the SWA score (Grade III or more), a wound culture should confirm the diagnosis, as described in the CDC definition (Centers for Disease Control and Prevention) [15].

### Secondary endpoints

Wound complications in both groups are assessed during the first 30 days postoperatively. Close attention will be paid to hematoma, femoral neuropathy, dehiscence, fluid discharge, stenotic/occlusive complications in the CFA and signs of infection.

When a *Staphylococcus aureus* SSI occurs (as the major contributor to all in the groin), preoperative isolates will be compared to postoperative SSI involved strains.

All patients will score their wound comfort or pain in a validated VAS score 1 day, and 2 weeks after surgery. Outcomes for open femoral versus percutaneous access will be compared.

Percutaneous access of the CFA is expected to inflict less surgical site infections in the groin compared to open surgical cutdown of the CFA. A lower incidence of infections and localized postoperative problems is expected to offset the additional costs of the percutaneous device. Still, the reduction in complaints needs to be quantified.

Follow-up will be performed until 1 year after the operation, according to the definition of a surgical site infection: “if implant (the endoprosthesis) is in place and the infection appears to be related to the operation” [15].

Recent studies suggest that certain patients may be colonized by multiple different *S. aureus* clones [16]. Also, these bacteria may hide intracellularly [17]. In case of a SSI, we hope to identify the initial localization of the causative microorganism. Therefore, preoperative cultures and punch biopsies will be used, respectively, to study the clonality of isolates by genotyping and to identify intracellular bacteria by fluorescence *in situ* hybridization (FISH) and fluorescence microscopy [18].

### Ethical considerations

Patients who meet the entry criteria will be fully informed about the trial and provided with a patient information and consent form. Patients willing to participate in the study will be included after signing the informed consent form. This study is being conducted in accordance with the principles of the Declaration of Helsinki and Good Clinical Practice guidelines. The protocol, site-specific informed consent forms, participant education and recruitment materials, and other requested documents were reviewed and approved by the Medical Ethics committee of Groningen (CMO 2013-365) and the local institutional board of each participating center (Table 2).

### Safety and quality control

Adverse events (AE) are defined as any undesirable experience occurring to a participant during the study, whether or not considered related to the investigational device. This definition includes events occurring during the hospital stay and up to 30 days of follow-up. Underlying disease that was present at the time of enrollment is not reported as an AE, but any increase in the severity of the underlying disease will be reported as an AE. All AEs will be monitored from the time of enrollment through the 30-day follow-up. AE’s will be recorded on the case record forms (CRFs). A description of the event, including the start date, end date, action taken and the outcome will be reported.

A severe adverse event (SAE) is any event leading to death, major amputation, prolonged hospitalization or renewed hospitalization due to complications and will be communicated to the Medical Ethical committee through “Toetsingonline.”

### Inclusion criteria

In order to be eligible to participate in this study, a subject must meet all of the following criteria:

1. More than 18 years of age.
2. Informed consent.
3. Physically and mentally capable of giving consent for participation and data storage.
4. diagnosed with an aneurysm of the abdominal aorta with a diameter of at least 55 mm (or growth of 5 mm or more in 6 months) suitable for bifurcated endovascular repair through two femoral access sites, without additional access needed.

### Exclusion criteria

Exclusion criteria are indicated as follows:

1. Patients with severe atherosclerosis at the access site (more than 50 % of the circumference of the common femoral artery).
2. Patients with previous common femoral artery surgery.
3. Patients with a documented infection at the time of operation.
4. Patients treated with an aorto-monoiliacal device implanted, followed by a femoro-femoral crossover.
5. Patients with an aneurysm necessitating more than two accesses (brachial or carotid) for repair.

### Recruitment

Patients more than 18 years of age and presenting at the outpatient clinic of participating hospitals, with an indication for bifurcated EVAR will be asked to participate. Patients will be informed about the purpose, and intervention of the study, its duration and the risks. Consent will be obtained with explanation and a signature on the Informed Consent Form. Patients have 1 week to consider their decision.

Withdrawal from the study is possible at any time for any reason and is without further consequences.

### Randomization

Randomization will be performed prior to operation. The operating physician or his/her assistant must draw a blinded envelop to allocate the patient to one of the two groups.

In the first group (group A), surgical access is achieved for the main body of the endoprosthesis. In the second group (group B), the Prostar XL™/Proglide™ device gives passage to the main device.

The allocation will be noted at the case report form (CRF) present in the envelope, which also contains a circular blade of 3 mm diameter, and 3 culture mediums (Thioglycollate medium USP, Mediaproducts, Groningen, Germany). Two cultures are taken after completion of the preoperative checklist and after administration of prophylactic antibiotics (1 g cefazolin, intravenously 15 to 60 min before surgery): one nose culture and one perineal culture is performed with a swab, rotated at the nasal and perineal site. The punch biopsy is collected from the right groin after disinfection, a circular blade of 3 mm is rotated and 2 skin biopsies are harvested. One punch biopsy is stored in formaldehyde; the second punch biopsy is stored in the third culture medium.

The culture media and formaldehyde storages have been coded in advance by the investigator, who prepared the content of the blinded envelop, and these samples will be sent to the laboratory of CERTE in an enclosed addressed envelope.

### Blinding

The operating team cannot be blinded for the allocation. Medical staff responsible for control visits and wound evaluation will be blinded for the passage of the main device or the contralateral leg. The scar of the Prostar XL™/Proglide™ device will, however, be smaller. This is an investigator-blinded study.

### Emergency unblinding

To maintain the overall quality and legitimacy of the clinical trial, code breaks should occur only in exceptional circumstances when knowledge of the actual treatment is absolutely essential for further management of the patient. If unblinding is deemed necessary, the investigator should use the system for emergency unblinding through contacting the supervising investigator in writing. The objective database manager, who supervises the codes, is not allowed to give access to the coded patients, unless the supervising investigator (C.J. Zeebregts), in a written memo, gives approval.

### Treatment details

#### Participant timelines

Participants are enrolled in the PiERO trial during their preoperative evaluation. Postoperative assessment is performed during the regular outpatient follow up.

#### Concomitant care

In case of an SSI, the wound must be opened, cultured and rinsed with clean water, as considered appropriate according to the treating physician. When infection persists or escalates, oral or intravenous antibiotics are administered. If a second operation is necessary to restrain an infection, this will be registered as an AE.

This trial has no prohibited concomitant medication.

#### Follow-up

Wound control is performed at 1 day, 2 weeks and 6 weeks after operation. Final evaluation will take place at 1 year after the procedure. This evaluation will be carried out for infection of the endoprosthesis, according to the definition [15]. The wound evaluation is carried out using the Southampton Wound Assessment score. Wound complications emerging in between measuring points (1 day, 2 weeks and 6 weeks) will also be recorded.

Three VAS scores (Visual Analog Scale) will be obtained before the operation, 1 day after the operation, and 2 weeks after the operation.

Scores are registered in the CRF (case report form) and collected by the objective database manager (Table 3).

**Table 3:** Timeline for participants

	Before operation	1 day after surgery	2 weeks after surgery	6 weeks after surgery	1 year after surgery
VAS score	X	X	X		
Wound assessment		X	X	X	X

VAS, visual analog scale

#### Data management

All forms, diskettes and tapes related to study data will be kept by the database manager. Access to the study data will be restricted. Values will be stored in an Excel sheet. Each additional value added to the database will be stored in consecutively numbered Excel documents, in a security-coded computer. A complete back up of the primary DCC database will be performed weekly and will be stored in locked files (data cannot be altered).

### Statistical analyses

#### Primary study parameter(s)

The occurrence of SSIs in both groups will be compared with a McNemar’s test and proportional hazards regression. Only two measuring points will be defined, but wound complications are expected to emerge also between these measuring points. A Kaplan Meier curve may be used to log incidences according to time. Both groups will be compared using the log rank test.

#### Secondary study parameter(s)

The number of wound complications in both groups will be compared as mentioned previously.

Patient characteristics will be controlled by multivariate analysis with respect to the duration of operation, left/right differences, preoperative bacterial colonization of the nose and the perineum, diabetes mellitus, body mass index, medication, smoking habits and device failure. Although identical groups are examined, we will be able to identify outliers.

Correlations between factors will be estimated using the Pearson correlation coefficient. Proportions of categorical variables are compared using Chi square and Fisher Exact tests, and means of continuous variables are compared using t tests.

The analysis of the primary endpoint (surgical site infection) will be based on an intention-to-treat principle by proportional hazards regression (visualized by Kaplan-Meier survival curves). Intention-to-treat implies that open access because of failed percutaneous access is not excluded from analysis but remains in the percutaneous group. Reasons why percutaneous access may not be successful are inability to obtain access to the arterial lumen with a needle, rupture of the threads of the Prostar XL™ device/2 Proglide™ devices, necessitating open repair and persistent bleeding despite successful use of the Prostar XL™ device/2 Proglide™ devices. The survival analysis is not affected by patient withdrawals (as they will be censored) provided that dropping out is unrelated to prognosis (Table 4). Other outcomes, such as VAS scores 2 weeks post-randomization, may be missing for patients who withdraw from the trial. We will actively seek out reasons for withdrawal and compare the reasons qualitatively.

**Table 4:** Number of included patients in the participating centers

Participating center	Number of included patients
- University Medical Center Groningen	13 patients included
- Isala Hospital Zwolle	19 patients included
- Medical Center Leeuwarden	14 patients included
- Wilhelmina Hospital Assen	8 patients included
- Scheper Hospital Emmen	1 patient included
Total:	55 patients included

#### Publication of data

The scientific integrity of the project requires that the data from all sites be analyzed study-wide and reported as such. Thus, an individual center is not expected to report the data collected from its center alone. All presentations and publications are expected to protect the integrity of the major objectives of the study.

Recommendations will be given by Abbott, and Abbott will be granted the nonexclusive right to use any publication or presentation resulting from the research.

Publications will be prepared under the responsibility of the principle investigator and the contributions of all authors will be specified. A copy of such publication will be provided to Abbott for its review and comment. The principle investigator will give due consideration to any comments made by Abbott. Changes will be incorporated only when Abbott will require protection of Abbott’s proprietary rights and interests. This will not interfere with the ethical responsibility to disseminate the trial results in an unbiased and timely manner; consequently, Abbott cannot prohibit publication.

## Discussion

Comparison of the incidence of SSIs in one patient may answer (an important part of) the question whether percutaneous closure devices deserve a role in the prevention of SSIs after EVAR. The cost of an SSI is estimated to be between US $10.000, and $20.000 per infection [19]. In pEVAR, a trend was noticed to reduce procedure and hospitalization times [10–12, 20]. The aim of this study is to contribute to the rationale of using percutaneous devices for femoral access in EVAR.

Analysis will be performed on an intention-to-treat basis. When a percutaneous access, for some reason, is not successful, open access will be obtained. Reasons why percutaneous access may not be successful are described in the Statistical Analysis section.

For all participants, cultures of the nose and the perineum will be taken preoperatively. In addition, two punch biopsies from the right groin will be obtained to evaluate the presence of microorganisms in deeper layers of the skin or intracellularly (by culture/molecular methods and PA research). The intention is to compare cultures and SSIs as it becomes more apparent that differences in hostility between bacterial species and/or clones exists [16, 17]. Therefore, the assumption was made, that a SSI does not only develop due to flaws in sterility [21], but also because some bacteria might be able to conceal themselves better in or on their host, than other bacteria. We know that bacteria can hide intracellularly [17, 22], and take over resistance genes from each other. Therefore, it might be interesting to compare *S. aureus* strains not causing SSIs, to those that do cause an SSI.

In conclusion, the PiERO trial is a multicenter randomized controlled clinical trial designed to show the consequences of using percutaneous access in EVAR surgery, when considering surgical site infections. In addition, patient comfort, estimated hospital stay and other wound complications are taken under consideration.

## Trial status

Patients have been included as follows in the various participating centers:

## Abbreviations

AE: adverse event
CERTE: the name of the infectious laboratory
CFA: common femoral artery
CMO: Commissie Mensgebonden Onderzoek = Commission Research on Humans
CRF: case report form
EVAR: endo-vascular aneurysm repair
FISH: fluorescence in situ hybridization
Fr.: French
Nm: Not mentioned
pEVAR: percutaneous endovascular aneurysm repair
PiERO: Percutaneous access in Endovascular Repair versus Open access
SAE: serious adverse event
SSI: surgical site infection
SWA-score: Southampton Wound Assessment score
VAS: Visual Analog Scale
